# The Role of Sulfur Dioxide in the Regulation of Mitochondrion-Related Cardiomyocyte Apoptosis in Rats with Isopropylarterenol-Induced Myocardial Injury

**DOI:** 10.3390/ijms140510465

**Published:** 2013-05-21

**Authors:** Hongfang Jin, Angie Dong Liu, Lukas Holmberg, Manman Zhao, Siyao Chen, Jinyan Yang, Yan Sun, Shanshan Chen, Chaoshu Tang, Junbao Du

**Affiliations:** 1 Department of Pediatrics, Peking University First Hospital, Xi’an Men Str. No. 1, West District, Beijing 100034, China; E-Mails: jinhongfang51@126.com (H.J.); manman8214908@163.com (M.Z.); siyaochen_nymaz@163.com (S.C.); yangjinyan_tg@163.com (J.Y.); yansun2008@gmail.com (Y.S.); s.s_chen@yahoo.com.cn (S.C.); 2 Department of Medical and Health Sciences, Linköping University, Linköping 58183, Sweden; E-Mails: angie.dongliu@gmail.com (A.D.L.); lukas.holmberg20@gmail.com (L.H.); 3 Laboratory of Molecular Cardiology, Ministry of Education, Beijing 100191, China; E-Mail: tangchaoshu@263.net.cn; 4 Department of Physiology and Pathophysiology, Health Sciences Center, Peking University, Beijing 100191, China

**Keywords:** isopropylarterenol, sulfur dioxide, myocardium, apoptosis, mitochondrial membrane potential

## Abstract

The authors investigated the regulatory effects of sulfur dioxide (SO_2_) on myocardial injury induced by isopropylarterenol (ISO) hydrochloride and its mechanisms. Wistar rats were divided into four groups: control group, ISO group, ISO plus SO_2_ group, and SO_2_ only group. Cardiac function was measured and cardiomyocyte apoptosis was detected. Bcl-2, bax and cytochrome c (cytc) expressions, and caspase-9 and caspase-3 activities in the left ventricular tissues were examined in the rats. The opening status of myocardial mitochondrial permeability transition pore (MPTP) and membrane potential were analyzed. The results showed that ISO-treated rats developed heart dysfunction and cardiac injury. Furthermore, cardiomyocyte apoptosis in the left ventricular tissues was augmented, left ventricular tissue bcl-2 expression was down-regulated, bax expression was up-regulated, mitochondrial membrane potential was significantly reduced, MPTP opened, cytc release from mitochondrion into cytoplasm was significantly increased, and both caspase-9 and caspase-3 activities were increased. Administration of an SO_2_ donor, however, markedly improved heart function and relieved myocardial injury of the ISO-treated rats; it lessened cardiomyocyte apoptosis, up-regulated myocardial bcl-2, down-regulated bax expression, stimulated mitochondrial membrane potential, closed MPTP, and reduced cytc release as well as caspase-9 and caspase-3 activities in the left ventricular tissue. Hence, SO_2_ attenuated myocardial injury in association with the inhibition of apoptosis in myocardial tissues, and the bcl-2/cytc/caspase-9/caspase-3 pathway was possibly involved in this process.

## 1. Introduction

Myocardial injury is a common pathophysiologic phenomenon in a variety of cardiovascular diseases [1,2]. Loss of cardiomyocytes through apoptosis is an important pathological change in myocardial injury. Thus, more and more researchers consider preventing cardiomyocyte apoptosis as a key therapy target of cardiovascular diseases [3]. However, the pathogenesis responsible for myocardial apoptosis has not been fully understood.

Though sulfur dioxide (SO_2_) had been regarded as a harmful air pollutant [4,5], it was also found that SO_2_ might be generated endogenously from sulfur-containing amino acid metabolism pathway in mammals [6]. Furthermore, endogenous SO_2_ production was detected in cardiovascular tissues and its regulatory effects on the cardiovascular structure and function were discovered, including lowering blood pressure, relaxing blood vessels and a negative inotropic effect in the heart [7–10]. Most interestingly, recent studies revealed that a change in endogenous SO_2_ production was associated with the development of many cardiovascular diseases [11–14].

In previous studies we found plasma and myocardial SO_2_ level was decreased in the rat model of ISO-induced myocardial injury. Administration of SO_2_ donor increased plasma and myocardial SO_2_ level and prevented myocardial injury. The mechanism was mainly related to promoting reactive oxygen species (ROS) scavengers, inhibiting the excessive endoplasmic reticulum stress, and restoring the intracellular calcium homeostasis [13–16]. The above pathological processes were all related with the survival and apoptosis of cardiomyocytes and deepened the knowledge of development of myocardial apoptosis. However, the mechanism by which endogenous SO_2_ affects cardiomyocyte apoptosis remains unclear.

It is well known that mitochondrial dysfunction is an important feature in apoptosis as well as a prominent factor associated with cell death and apoptosis [17]. Bcl-2 family proteins were regarded to govern mitochondrial outer membrane permeability and determine the outcome of an intrinsic apoptotic process initiated by the release of cytc and apoptotic factors from the mitochondria [18]. Liang *et al*. reported that SO_2_ markedly attenuated ISO-induced mitochondrial swelling and deformation [13]. Therefore, the present study was undertaken to investigate the inhibitory effect of SO_2_ on cardiomyocyte apoptosis in the development of ISO-induced myocardial injury, and explore its mechanism in relation to the preservation of mitochondrial function.

## 2. Results

### 2.1. SO_2_ Improved Rat Heart Function in ISO-Treated Rats

**Figure 1:**
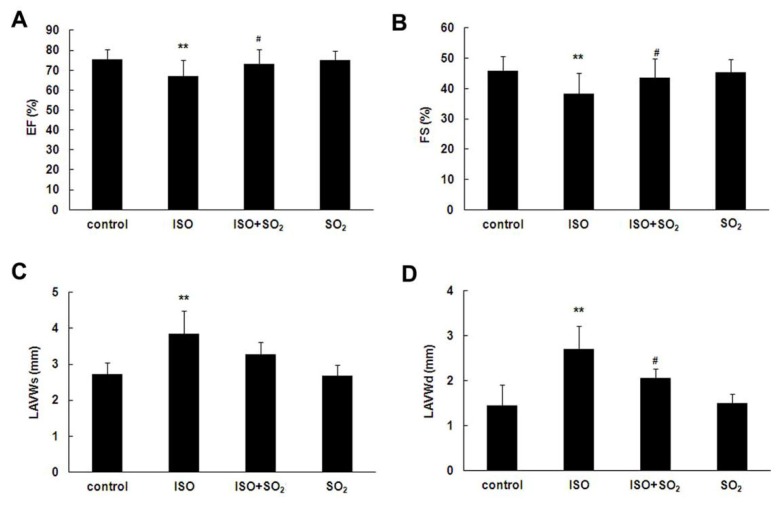
Echocardiographic determination of cardiac function in rats. At day 8 of the experiment, echocardiographic detection was conducted on the rats. Echocardiographic quantitative analysis software was used to detect the values of 3 continuous cardiac cycles. (**A**) Change of left ventricular ejection fraction (EF); (**B**) Change of left ventricular fractional shortening (FS); (**C**) Change of systolic left ventricular anterior wall thickness (LVAWs); (**D**) Change of diastolic left ventricular anterior wall thickness (LVAWd). ISO: isopropylarterenol; SO_2_: sulfur dioxide; **p < 0.01 *vs*. control group, ^#^*p* < 0.05 *vs*. ISO group.

ISO at 20 mg/kg daily via subcutaneous injection for 7 continuous days induced a marked left ventricular dysfunction in rats demonstrated by echocardiographic analysis on the morning of day 8 (Figure 1). Compared with the control group, left ventricular ejection fraction (EF) and fraction shortening (FS) of rats of the ISO group were reduced (both *p* < 0.01), whereas systolic left ventricular anterior wall thickness (LVAWs) and diastolic left ventricular anterior wall thickness (LVAWd) were increased (both *p* < 0.01). Administration of SO_2_ donor at 85 mg/kg daily by intraperitoneal injection for 7 continuous days improved EF and FS of the ISO-treated rats, and reduced LVAWd (both *p* < 0.05). There were no differences, however, in EF, FS, LVAWs and LVAWd, between SO_2_ only group and control group.

### 2.2. SO_2_ Relieved Myocardial Injury in ISO-Treated Rats

**Figure 2:**
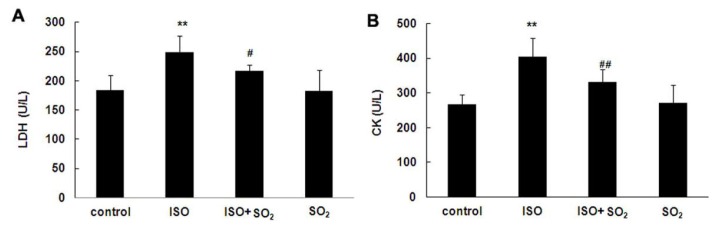
Plasma lactate dehydrogenase (LDH) and creatine kinase (CK) activity in rats. (**A**) Change of plasma LDH activity; (**B**) Change of plasma CK activity. ISO: isopropylarterenol; SO_2_: sulfur dioxide; **p < 0.01 *vs*. control group; ^#^*p* < 0.05; ^##^*p* < 0.01 *vs*. ISO group.

Plasma lactic dehydrogenase (LDH) and creatine kinase (CK) activities in 4 groups of rats were examined within 24 h after the samples were collected. Figure 2 showed that plasma LDH and CK activity in the rats of ISO group were increased compared with those of control group (both *p* < 0.01). Administration of SO_2_ donor for 7 continuous days decreased the plasma LDH and CK activities (both *p* < 0.05). There were no differences, however, in plasma LDH and CK activities between SO_2_ only group and control group.

### 2.3. SO_2_ Inhibited Cell Apoptosis Induced by ISO in Rat Myocardial Tissues

**Figure 3:**
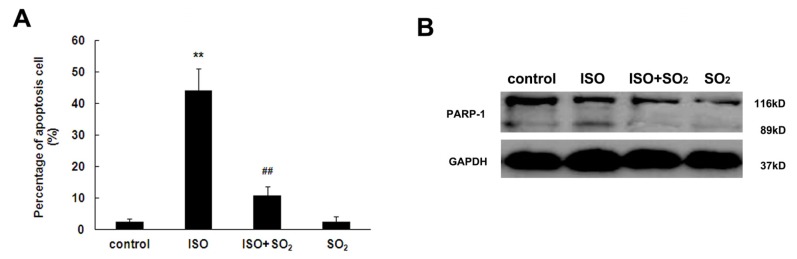

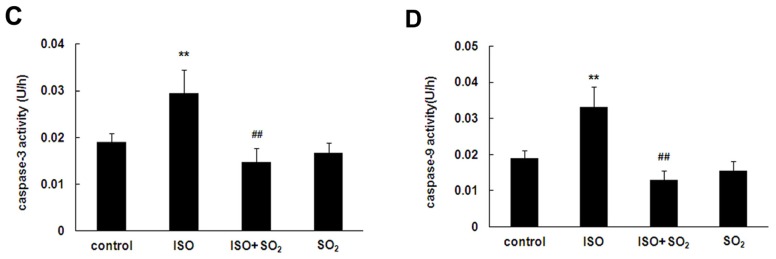
Change in cell apoptosis, caspase-9 and caspase-3 activities in left ventricular tissues of rat. (**A**) The difference among the cell apoptosis in myocardial tissues of the four groups of rats was observed by terminal deoxynucleotidyl transferase-mediated dUTP-biotin nick end labeling (TUNEL) assay. The percentage of apoptotic cells in myocardial tissues was calculated by semi-quantitative counting; (**B**) Poly(ADP-ribose) polymerase-1 (PARP-1) cleavage in myocardial tissues of rat; (**C**) Caspase-3 activity in myocardial tissues of rat; (**D**) Caspase-9 activity in myocardial tissues of rat. ISO: isopropylarterenol; SO_2_: sulfur dioxide; **p < 0.01 *vs*. control group, ^##^*p* < 0.01 *vs*. ISO group.

Cardiomyocyte apoptosis in the left ventricular tissue of rat was evaluated by detecting terminal deoxynucleotidyl transferase-mediated dUTP-biotin nick end labeling (TUNEL)-positive cells in the paraffin embedded tissue slice, and examining poly(ADP-ribose) polymerase-1 (PARP-1) cleavage, as well as caspase-3 and caspase-9 activities in left ventricular tissues. As shown in Figure 3A, the percentage of TUNEL-positive cells in left ventricular tissues was increased in rats of the ISO group, whereas SO_2_ decreased the percentage of TUNEL-positive cells by 75% (*p* < 0.01). Similarly, SO_2_ reduced ISO-induced PARP cleavage, and caspase-3 and caspase-9 activities in rat left ventricular myocardium (Figure 3B–D). There were also no differences in the percentage of TUNEL-positive cells, cleavage of PARP-1, myocardial caspase-3 and caspase-9 activities in rat left ventricular tissues between SO_2_ only group and control group.

### 2.4. SO_2_ Blocked the Effect of ISO on Myocardial bcl-2 and Bax Expression

**Figure 4:**
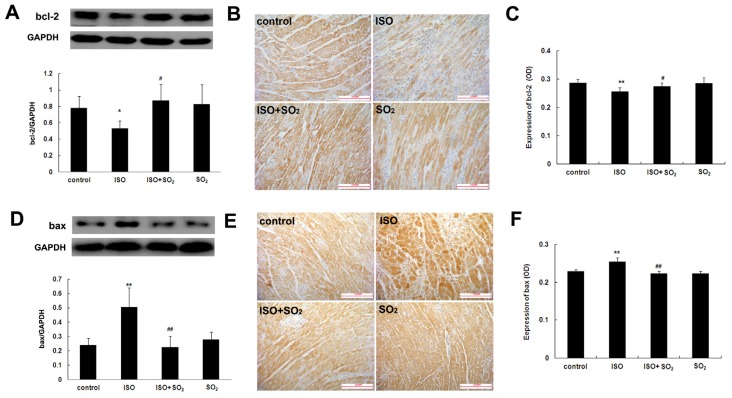
Bcl-2 and bax protein expression in rat left ventricular tissues. (**A**) Bcl-2 protein expression in rat myocardial tissues was detected by western blot; (**B**) and (**C**) Bcl-2 protein expression in rat myocardial tissues was detected by immunohistochemistry; (**D**) Bax protein expression in rat myocardial tissues was detected by western blot; (**E**) and (**F**) Bax protein expression in rat myocardial tissues was detected by immunohistochemistry. ISO: isopropylarterenol; SO_2_: sulfur dioxide; **p < 0.01 *vs*. control group, ^##^*p* < 0.01, ^#^*p* < 0.05 *vs*. ISO group.

Western blot assay showed that compared with the control group, left ventricular tissue bcl-2 protein expression of the ISO group was significantly reduced (*p* < 0.01). However, SO_2_ increased left ventricular tissue bcl-2 protein expression in the ISO-treated rats (*p* < 0.05). Compared with the control group, left ventricular tissue bcl-2 protein expression in rats of the SO_2_ group did not significantly change (Figure 4A). Immunohistochemical assay showed that compared with the control group, myocardial bcl-2 protein expression of the rats in the ISO group was significantly reduced (*p* < 0.01); compared with the ISO group, myocardial bcl-2 protein expression of rats in the ISO plus SO_2_ group was significantly increased (*p* < 0.05); but compared with the control group, myocardial bcl-2 protein expression of rats in the SO_2_ group had no significant change (*p* > 0.05) (Figure 4B,C).

Western blot assay showed that compared with the control group, left ventricular tissue bax protein expression in rats of the ISO group significantly rose (*p* < 0.01). However, SO_2_ attenuated the increased myocardial bax protein expression of ISO-treated rats (*p* < 0.05). Compared with the control group, myocardial bax protein expression in rats of the SO_2_ group did not significantly change (Figure 4D). Immunohistochemical assay showed that compared with the control group, myocardial positive bax protein signals were obviously strong in rats of the ISO group (*p* < 0.01). Compared with the ISO group, however, myocardial bax protein expression of rats in the ISO plus SO_2_ group was reduced (*p* < 0.01). Compared with the control group, myocardial bax protein expression of rats in the SO_2_ only group had no significant change (*p* > 0.05) (Figure 4E,F).

### 2.5. SO_2_ Antagonized the Inhibition of Myocardial Mitochondrial Membrane Potential by ISO in Rats

**Figure 5:**
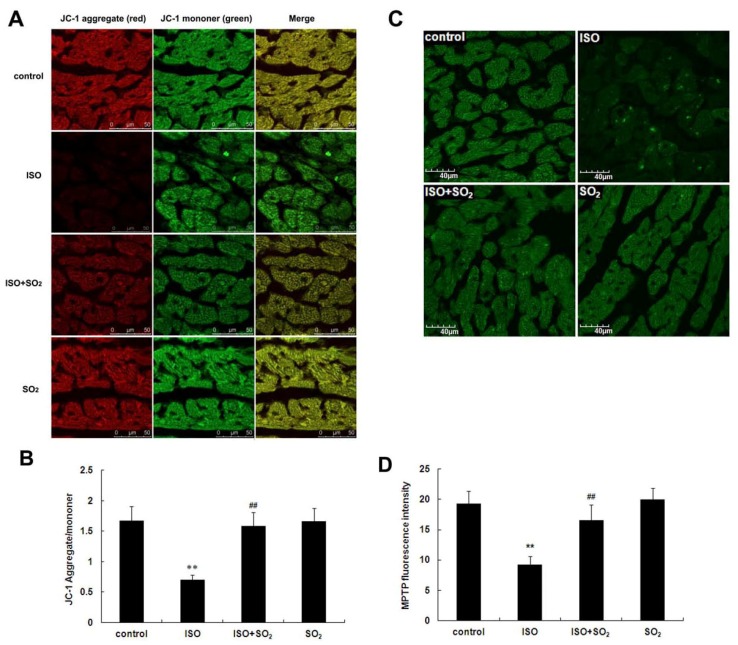
Changes in mitochondrial membrane potential and mitochondrial permeability transition pore (MPTP) opening in rat myocardial tissues. (**A**) The change in mitochondrial membrane potential was detected with JC-1 fluorescent probe by laser confocal microscopy. Red color represented JC-1 aggregate and green color represented JC-1 monomer; (**B**) Summarized data for the relative changes in JC-1 fluorescence; (**C**) The change in MPTP opening was detected with calcein-AM as a fluorescence indicator by laser confocal microscopy; (**D**) Summarized data for the relative changes in calcein fluorescence. ISO: isopropylarterenol; SO_2_: sulfur dioxide; **p < 0.01 *vs*. control group, ^##^*p* < 0.01 *vs*. ISO group.

Cardiomyocyte mitochondrial membrane potential in rats of the ISO group was significantly reduced compared to the control group (*p* < 0.01). SO_2_ increased the cardiomyocyte mitochondrial membrane potential in the ISO-treated rats (*p* < 0.01). Compared with the control group, the cardiomyocyte mitochondrial membrane potential of the rats in the SO_2_ only group did not change obviously (Figure 5A,B).

### 2.6. SO_2_ Inhibited Myocardial Mitochondrial Permeability Transition Pore (MPTP) Opening Induced by ISO

Compared with the control group, myocardial fluorescence intensity of rats in the ISO group was significantly weakened, suggesting the opening of MPTP. When the rats were treated with SO_2_, cardiomyocyte fluorescence intensity of rats in the ISO plus SO_2_ group was significantly strengthened, suggesting the closing of MPTP. Compared with the control group, myocardial fluorescence intensity of rats of the SO_2_ only group did not alter (*p* > 0.05) (Figure 5C,D).

### 2.7. SO_2_ Antagonized the Increase in Myocardial Cytc Protein Expression Induced by ISO

**Figure 6:**
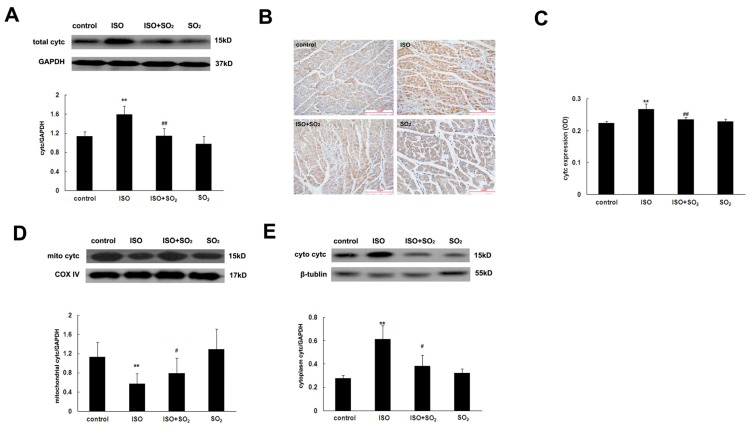
Cytochrome c (cytc) protein expression and distribution in rat left ventricular tissues. (**A**) Cytc expression in rat myocardial tissue detected by western blot; (**B**) Representative images of cytc expression in rat cardiomyocytes detected by immunohistochemical analysis; (**C**) Semi-quantitative analysis for the relative changes in cytc expression by immunohistochemistry; (**D**) Cytc expression in the mitochondrial fraction of rat myocardial tissue detected by western blot; (**E**) Cytc expression in the cytosolic fraction of rat myocardial tissue detected by western blot. ISO: isopropylarterenol; SO_2_: sulfur dioxide; COX IV: cytochrome c oxidase IV. **p < 0.01 *vs*. control group; ^##^*p* < 0.01, ^#^*p* < 0.05 *vs*. ISO group.

Western blot showed that compared with the control group, left ventricular tissue cytc protein expression of rats in the ISO group was increased (*p* < 0.01). SO_2_ could markedly reduce the increased myocardial cytc protein expression in rats of the ISO plus SO_2_ group (*p* < 0.01). Compared with the control group, left ventricular tissue cytc protein expression of rats in the SO_2_ only group did not significantly change (Figure 6A).

Immunohistochemical analysis showed that compared with the control group, the signals of myocardial cytc protein of rats in the ISO group were significantly strengthened (*p* < 0.01). However, compared with the ISO group, myocardial cytc protein signals were significantly weakened in rats of the ISO plus SO_2_ group (*p* < 0.01). The signals did not differ between the control group and the SO_2_ only group (*p* > 0.05) (Figure 6B,C).

### 2.8. SO_2_ Inhibited the ISO-Induced Release of Cytc from the Mitochondrion into the Cytoplasm of Cardiomyocytes

Western blot showed that compared with the control group, cytc content in the cytoplasm of rats in the ISO group was increased but cytc content in the mitochondrion decreased (*p* < 0.01). Compared with the ISO group, cytc content in the cytoplasm of rats in the ISO plus SO_2_ group was reduced but cytc content in the mitochondrion increased (*p* < 0.01). Compared with the control group, both cytc content in cytoplasm and cytc content in mitochondrion of rats in the SO_2_ only group did not change (*p* > 0.05) (Figure 6D,E).

## 3. Discussion

Exploring options for cardioprotection from myocardial injury attracts a great amount of attention in cardiovascular research and clinical trials. Today, drugs targeting the renin-angiotensin system and nitric oxide are the common cardiovascular agents of clinical utility [19,20]. In an attempt to examine other signaling pathways, Geng *et al*. discovered that the endogenous hydrogen sulfide, an end product of sulfur-containing amino acid metabolic pathways, contributed to the cardioprotection of ISO-induced myocardial injury. Administration of a hydrogen sulfide donor effectively protected cardiomyocytes and their contractile activity, at least in part by its direct scavenging of oxygen-free radicals and reducing the accumulation of lipid peroxidations [21].

Actually, SO_2_, another end product of sulfur-containing amino acid metabolic pathways, was previously regarded as an atmospheric pollutant [4,5]. Recently, our research team found that there was an endogenous SO_2_ generation pathway in the cardiovascular system [7,8] and that SO_2_ played important cardiovascular physiological [7,9,10] and pathophysiological roles [11–16]. In particular, our previous studies [13] showed that in the model of myocardial injury induced by ISO, endogenous myocardial SO_2_ generation was significantly reduced. In the present study, cardiac EF had a statistically significant decrease in ISO-treated rats compared with that of control rats though the decrease appears small. Other cardiac functional indexes, such as FS, and plasma CK and LDH levels also showed marked changes, indicating the presence of myocardial injury. After an SO_2_ donor was supplemented, myocardial injury was greatly alleviated, which suggested that the endogenous SO_2_ pathway down-regulation was involved in the pathogenesis of ISO-induced myocardial injury and that SO_2_ had an important cardioprotective effect. However, the mechanisms by which SO_2_ protects against myocardial injury remain unclear.

Cardiomyocyte apoptosis is involved in the development of cardiomyopathy, myocardial ischemia reperfusion and heart failure [22–24]. Therefore, the present study was designed to investigate the mechanism responsible for cardioprotection by SO_2_ from the viewpoint of cell apoptosis. The apoptosis of cardiomyocytes was represented by detecting TUNEL-positive cell, cleaved PARP, caspase-3 and caspase-9 activities. PARP is a substrate for caspase-3, and cleaved PARP-1 has been shown to be an important marker for apoptosis [25]. Caspase-9 was activated by cytc released from mitochondrion and subsequently activated the caspase cascade reaction and finally activated caspase-3 to cause cell apoptosis [26]. The results of the present study showed that the percentage of apoptotic cells in the left ventricular tissues of rats in the ISO group was increased, and cleavage of PARP-1, casapase-3 and caspase-9 activites were also enhanced, whereas the administration of a SO_2_ donor both reduced apoptotic cells in left ventricular tissues of ISO-treated rats and reduced the cleavage PARP-1, casapase-3 and caspase-9 activities, indicating that SO_2_ could inhibit cardiomyocyte apoptosis in left ventricular tissues induced by ISO. Further studies are needed to investigate the possible mechanisms by which SO_2_ inhibits cardiomyocyte apoptosis.

The bcl-2/cytc/caspase-9/caspase-3 apoptosis pathway is a common apoptotic pathway [27]. Bcl-2 family proteins are known to determine the outcome of an intrinsic apoptotic process initiated by the release of cytc and apoptotic factors from the mitochondria. Bcl-2 and bax are a pair of interacting proteins in the bcl-2 family. Bcl-2 mainly plays an anti-apoptotic role [18,28], while bax mainly plays a pro-apoptotic role [29,30]. In this experiment, compared with the control group, bcl-2 protein expression in rat myocardial tissues of the ISO group was significantly down-regulated, and bax protein expression was significantly up-regulated, indicating that the anti-apoptotic ability of cardiomyocytes was lowered and pro-apoptotic ability increased. SO_2_ administration to the rats treated with ISO, however, attenuated bax up-regulation and prevented bcl-2 down-regulation, indicating that SO_2_ could prevent the imbalance of cardiomyocyte bcl-2/bax and the subsequent apoptosis induced by ISO. Regarding the mechanism by which SO_2_ regulated bcl-2 expression, we supposed that the PI3K/Akt and/or ROS pathways might be the possible target(s) according to the following studies: Wang *et al*. reported that bax/bcl-2 expression in the HepG2 cell was promoted with LY294002 (an inhibitor of PI3K) intervention but blocked by PI3K activator and NAC (a ROS scavanger) pretreatment, suggesting that PI3K/Akt activation might decrease bax/bcl-2 ratio and ROS might increase bax/bcl-2 ratio [31]; Zhao *et al*. and Liang *et al*. reported that SO_2_ could activate the PI3K/Akt pathway and decrease the ROS level in the myocardial injury [13,32].

The downstream molecule of the bcl-2 protein family is mainly cytc. Under physiological condition, cytc is mainly present in the mitochondrion, not inducing apoptosis. When apoptotic signals are received, cytc is released from the mitochondrion into the cytoplasm to induce apoptosis. The release of cytc from the mitochondrion into the cytoplasm is the key step of apoptotic pathways [33]. Therefore, we used the fluorescence probe to detect the changes in mitochondrial membrane potential and the opening status of MPTP. Western blot and immunohistochemical methods were used to detect the change in the total amount of cardiomyocyte cytc, and western blot was also used to detect the release change of cytc from the mitochondrion into cytoplasm. The results showed that compared with the control group, the cardiomyocyte mitochondrial membrane potential of the ISO group was decreased, the MPTP opening was promoted, and the mitochondrial cytc was decreased but cytoplamic cytc was increased, indicating that ISO damaged mitochondrial membrane potential, induced the opening of MPTP, and then promoted cytc release from the mitochondrion into the cytoplasm via the opened MPTP. In contrast, SO_2_ increased cardiomyocyte mitochondrial membrane potential and reduced the mitochondrion MPTP opening of the ISO-treated rats. Consequently, SO_2_ prevented cytc release from the mitochondrion to the cytoplasm, indicating that SO_2_ could inhibit ISO-induced mitochondrial membrane structural and functional damage and mitochondrial cytc release.

In addition, Na_2_SO_3_/NaHSO_3_ was used as SO_2_ donor (85 mg/kg), according to previous studies [12,13]. The SO_2_ only group was set in this experiment where the SO_2_ donor was administered to control rats. As a result, there were no significant differences in heart function, myocardial injury, apoptosis in the myocardial tissues, and mitochondrion function between the SO_2_ only group and the control group, suggesting that administration of a SO_2_ donor (intraperitoneal injection of 85 mg/kg for 7 continuous days) had no cardiac side effects.

Taken together, this study demonstrated that the SO_2_ donor antagonized myocardial injury induced by ISO, improved heart function and played a cardioprotective role. SO_2_ inhibited ISO-induced cardiomyocyte apoptosis, which was possibly one of the myocardial protective mechanisms of SO_2_. SO_2_ promoted bcl-2 expression, inhibited bax expression, stimulated mitochondrial membrane potential, inhibited mitochondrion MPTP opening, inhibited the release of cytc from mitochondrion into cytoplasm, and inhibited the activation of caspase-9 and caspase-3, thus exerting an anti-apoptotic function. The above-mentioned results thus deepen the understanding of the mechanisms responsible for cardioprotection by SO_2_.

Although bcl-2 family protein expression and subcellular translocation are the major regulatory processes involved in the mitochondria-mediated apoptosis pathway, other factors and pathological processes are also involved in the mitochondria-mediated apoptosis, for example, ROS and endoplasmic reticulum stress [34,35]. Loor *et al*. demonstrated that oxidant stress caused release of calcein to the cytosol during ischemia, a response that was inhibited by chemically diverse antioxidants, or over-expression of Mn-super oxide dismutase, suggesting that mitochondrial oxidant stress caused MPTP opening [34]. Cunha *et al*. elucidated that a crosstalk between lipotoxic ER stress and the mitochondrial pathway of apoptosis caused β-cell death in diabetes [35]. Our previous study indicated that SO_2_ protected against ISO-induced myocardial injury associated with increased myocardial antioxidant capacity and had inhibitory effect on the endoplasmic reticulum stress in rats [13,14]. Therefore, in addition to Bcl-2 pathway, the possibility that SO_2_ protected the mitochondria function via scavenging ROS or excessively activated endoplasmic reticulum stress was worthy of further investigation.

## 4. Experimental Section

### 4.1. Animal Model

Two month old male Wistar rats with body weights ranging from 200 to 250 g were purchased from Vital River Laboratory Animal Center [Certificate number: SCXK (Jing) 2011-0011] and bred in the Experimental Animal Center of Peking University Health Science Center. A total of 39 rats were divided into four groups: control group (10 rats), ISO group (9 rats), ISO plus SO_2_ group (10 rats) and SO_2_ only group (10 rats). The control group received intraperitoneal injections with the equivalent amount of saline daily for 7 continuous days, the ISO group received subcutaneous injections with 20 mg/kg of ISO daily for 7 continuous days, the ISO plus SO_2_ group received intraperitoneal injections with 20 mg/kg of ISO daily for 7 continuous days and intraperitoneal injections with 85 mg/kg of Na_2_SO_3_/NaHSO_3_ for 7 days, and the SO_2_ only group received intraperitoneal injections with 85 mg/kg of Na_2_SO_3_/NaHSO_3_ daily for 7 continuous days. The four groups of rats fasted on the evening of the 7th day. The Ethical Committee for Animal Studies at the Peking University First Hospital approved the study.

### 4.2. Echocardiography Analysis

At day 7 of the experiment, all rats fasted in the evening of the latest administration. The next morning, echocardiography detection was conducted on the rats with Visual Sonics Vevo 770 Imaging System (Toronto, Canada). Inhalation anesthesia was conducted with isoflurane and oxygen gas mixture (isoflurane concentration of 3%) on the rats. The rats were then kept in a supine position, a 17.5 Hz two-dimensional cardiography scanner probe was used to conduct level scanning at the left sternal border papillary muscle of the rats and the images were saved. Echocardiography quantitative analysis software was used to detect the values of 3 continuous cardiac cycles, and the mean value was obtained. Main indicators included left ventricular EF, FS, LVAWs and LVAWd [36].

### 4.3. Specimen Collection

After the rats woke up after echocardiography, they were anaesthetized with 12% urethane (10 mL/kg). Plasma samples were obtained from the abdominal aorta using a heparinized syringe and centrifuged at 3000 rpm for 20 min. Cardiac apex tissues were fixated in 4% paraformaldehyde for 8 h and dehydrated in 20% sucrose phosphate buffer for 24 h. Subsequently, one part of the tissue was embedded in paraffin, and sliced into sections with the thickness of 7 μm. The other part of the tissue was embedded in OCT, and sliced into frozen sections with the thickness of 7 μm. The remaining left ventricular tissues were coded, rapidly frozen in liquid nitrogen and stored at −70 °C.

### 4.4. Detection of Myocardial Enzymatic Activity in Plasma

The activities of LDH and CK were measured using an Automated Enzymology Instrument (Hitachi 7080, Hitachi Ltd., Tokyo, Japan) [13].

### 4.5. TUNEL Assay

The paraffin-fixed sections were dewaxed and rehydrated with dimethylbenzene and gradient ethanol. Apoptotic cells were detected with the TUNEL kit following the manufacturer’s instructions (G3250, Roche, Nutley, NJ, USA) [37]. Briefly, the slide underwent fixation with 4% formaldehyde, permeabilization with 20 μg/mL proteinase K, repeated fixation with 4% formaldehyde, equilibration with equilibration buffer and labeling with TdT reaction mixture. Finally, the reaction was ended with 2× SSC and the slides were counterstained with hematoxylin, and the labeled cells were analyzed under a light microscope. The percentage of apoptotic cells was calculated as a ratio of the number of TUNEL-positive cells to the total number of cells. Cells in eight different randomized fields in the myocardium were counted.

### 4.6. Isolation of Mitochondrial and Cytosolic Fraction

Two hundred micrograms of left ventricular tissue were homogenized in 1 mL of isolation buffer (70 mM sucrose, 190 mM mannitol, 20 mM HEPES, 0.2 mM EDTA, 200 μM sodium orthovanadate, 10 μg/mL aprotinin, 10 μg/mL leupeptin, 0.5 mM *p*-nitrophenyl phosphate, and 1 mM PMSF). After homogenization, 70 μL of the homogenate were added to 400 μL of RIPA buffer (150 mM NaCl, 20 mM Tris·HCl pH 7.5, 0.1% SDS, 1% Triton X-100, 1 mM EDTA, 200 μM sodium orthovanadate, 10 μg/mL aprotinin, 10 μg/mL leupeptin, 0.5 mM *p*-nitrophenyl phosphate, and 1 mM PMSF), and this whole homogenate fraction was saved for western blot analysis. The remainder homogenate was centrifuged at 600× *g* for 10 min to remove nuclei and myofibrils. The resulting supernatant was centrifuged at 5000× *g* for 15 min, producing a pellet enriched in mitochondria and a supernatant. The pellet was washed twice with 1 mL of isolation buffer and then resuspended in 400 μL of RIPA buffer to produce the final mitochondrial fraction. The supernatant was centrifuged at 100,000*g* for 60 min. The resulting supernatant was the final cytosolic fraction. Cytochrome c oxidase IV (COX IV) and β-tubulin were detected by western blot as the markers of mitochondrial and cytosolic fraction, respectively.

### 4.7. Western Blot Assay

Expressions of bcl-2, bax and cytc as well as PARP-1 cleavage in rat left ventricular tissues were detected using western blot [8]. The BCA method was used to detect the protein concentration. The protein sample of 40 μg was taken, and SDS-PAGE electrophoresis of 10% separation gel was conducted. The separated proteins were electrophoretically transferred onto a nitrocellulose membrane for 3 h at 200 mA. The membranes were blocked with 5% dried skimmed milk for 1 h. Subsequently, the primary antibodies to bcl-2, bax, cytc and PARP-1 (sc-492, sc-493, sc-13156 and sc-1561, Santa Cruz, CA, USA) (diluted with PBS at 1:2000, 1:2000, 1:4000 and 1:1000, respectively) were added and incubated at 4 °C overnight. After washing, the second antibody was added and incubated for 1 h at room temperature. After washing, LumiGLO chemiluminescence reagent was used for reaction and exposure was carried out. At last, grey scanning was conducted for the bands, and grey level was quantified using an AlphaImager (San Leandro, CA, USA). Also, a semi-quantitative analysis was conducted for the corresponding protein expression.

### 4.8. Immunohistochemical Staining Method

The paraffin-fixed slide was dewaxed, rehydrated with dimethylbenzene and gradient ethanol (100%, 95% and 70%), incubated with 3% H_2_O_2_ for 10 min at room temperature, digested in pepsin for 10 min at 37 °C and blocked in goat serum for 30 min at 37 °C. Specific primary antibodies to bcl-2, bax and cytc (sc-492, sc-493 and sc-13156, Santa Cruz, CA, USA) (diluted with PBS at 1:200, 1:200 and 1:400, respectively) were separately added and incubated overnight at 4 °C. HRP-conjugated anti-rabbit IgG was used as a secondary antibody. Finally, the slide was incubated with DAB buffer at room temperature in a humidified and dark chamber, and then counterstained with hematoxylin. The brown granules in the cardiomyocytes observed under microscope were defined as the positive signals. For negative controls, sections were processed as above except that the primary incubation was performed with non-immune goat serum instead of primary antibodies. Leica Q550image analysis software (Leica, Cambridge, UK) was used to acquire images and analyze optical density values. For each section, 4 different fields were selected randomly and the mean was obtained [8].

### 4.9. Mitochondrial Membrane Potential Detection

Mitochondrial membrane potential was measured with a unique cationic dye of 5,5′,6,6′-tetrachloro1,1′,3,3′-tetraethyl benzimidazol carbocyanine iodide (JC-1) (GMS10013.6, Genmed Scientific Inc., Shanghai, China). In live cells, the mitochondria appear red due to the aggregation of accumulated JC-1, which have absorption/emission maxima of 585/590 nm (red). In apoptotic and dead cells, the dye remains in its monomeric form, which has absorption/emission maxima of 510/530 nm (green). The ratio of JC-1 aggregate (red) to monomer (green) intensity was calculated. A decrease in this ratio was interpreted as a decrease in the mitochondrial membrane potential, whereas an increase in this ratio was interpreted as a gain in the mitochondrial membrane potential [38]. Briefly, 100 μL of working solution of JC-1 dye was added to the frozen slide at 37 °C for 20 min in darkness. The excess dye was removed by washing with JC-1staining buffer and then rinsed three times with PBS. Observations were made immediately using a laser confocal scanning microscope (Leica, Cambridge, UK). For each slide, 4 different fields were selected randomly to acquire images and the average intensity of red and green fluorescence was determined.

### 4.10. MPTP Opening Detection

MPTP opening was determined with calcein-AM in the presence of cobalt chloride using the commercial MPTP fluorescence assay kit (GMS10095, Genmed Scientific Inc., Shanghai, China). When calcein gathered in the mitochondrion, it presented green fluorescent staining. When it was released into the cytoplasm via the opening of MPTP, fluorescent quenching occurred [34]. The frozen section was incubated with calcein-AM (1.0 μmol/L) and cobalt-chloride (1.0 mmol/L), resulting in mitochondrial localization of calcein fluorescence. MPTP opening was indicated by a reduction in mitochondrial calcein signal and was measured over 4 randomly chosen areas in each section under confocal microscope (emitting at 488 nm and detecting at 505 nm) (Leica, Cambridge, UK).

### 4.11. Caspase-9 Activity Assay

Caspase-9 activity in rat myocardial tissue was detected using the tissue caspase-9 colorimetric activity assay kit (GMS50037.2, Genmed Scientific Inc., Shanghai, China). The assay was based on spectophotometric detection of the chromophorep-nitroaniline (pNA) after cleavage from the labeled substrate Ac-LEHD-pNA [39]. The free pNA could be quantified using a microtiter plate reader at 405 nm. Comparison of the absorbance of pNA from an apoptotic sample with an un-induced control allowed determination of the fold increase in caspase-9 activity. Briefly, myocardial tissue was grounded in liquid nitrogen, homogenized with chilled tissue lysis buffer, incubated on ice for 10 min, and centrifuged for 5 min in a microcentrifuge (16,000× *g*). The supernatant (cytosolic extract) was collected to a fresh tube on ice. The protein concentration was assayed for each sample. The assay mixture was prepared in a 96-well plate and samples were incubated for 1 h at 37 °C. Finally, samples (at 405 nm) were read in a microtiter plate reader (Bio-rad, Hercules, CA, USA). Fold-increase in caspase-9 activity could be determined by comparing the OD reading from the induced apoptotic sample with the level of the un-induced control.

### 4.12. Caspase-3 Activity Assay

Caspase-3 activity in rat myocardial tissue was detected using the tissue caspase-3 colorimetric activity assay kit (GMS50029.2, Genmed Scientific Inc., Shanghai, China). The assay was based on spectophotometric detection of the pNA after cleavage from the labeled substrate Ac-DEVD-pNA [40]. The free pNA could be quantified using a microtiter plate reader at 405 nm. Comparison of the absorbance of pNA from an apoptotic sample with an un-induced control allowed determination of the fold increase in caspase-3 activity. The protocol was the same as the protocol of caspase-9 activity assay.

### 4.13. Statistical Analysis

Data are presented as mean ± SD. Statistical analysis was conducted with SPSS 13 software (SPSS Software, Inc., Chicago, IL, USA) using one-way ANOVA followed by either LSD test or Tamhane test according to the homogeneity of variance test, *p* < 0.05 was considered significant.

## 5. Conclusion

In summary, SO_2_ donor inhibited ISO-induced myocardial injury and heart dysfunction. The mechanism by which SO_2_ inhibited ISO-induced cardiomyocyte apoptosis might involve promoting bcl-2 expression, decreasing bax expression, stimulating mitochondrial membrane potential, preventing mitochondrion MPTP opening, and then inhibiting the release of cytc from mitochondrion into cytoplasm, inhibiting the activation of caspase-9 and caspase-3, thus exerting an anti-apoptotic function. The results would deepen the knowledge of the cardioprotection by SO_2_.
